# Supplementary material for: The interactome of intact mitochondria by cross-linking mass spectrometry provides evidence for coexisting respiratory supercomplexes
Source: Mol Cell Proteomics. 2017 Dec 8;17(2):216–32. doi: 10.1074/mcp.RA117.000470 (PMC5795388; doi:10.1074/mcp.RA117.000470)
Supplement: Supplemental Data [file supp_RA117.000470_133922_0_supp_23978_fzffwf.zip › spectra_annotation/mito_DR_spectra_annotation/323-1-18-1-11-1.pdf]

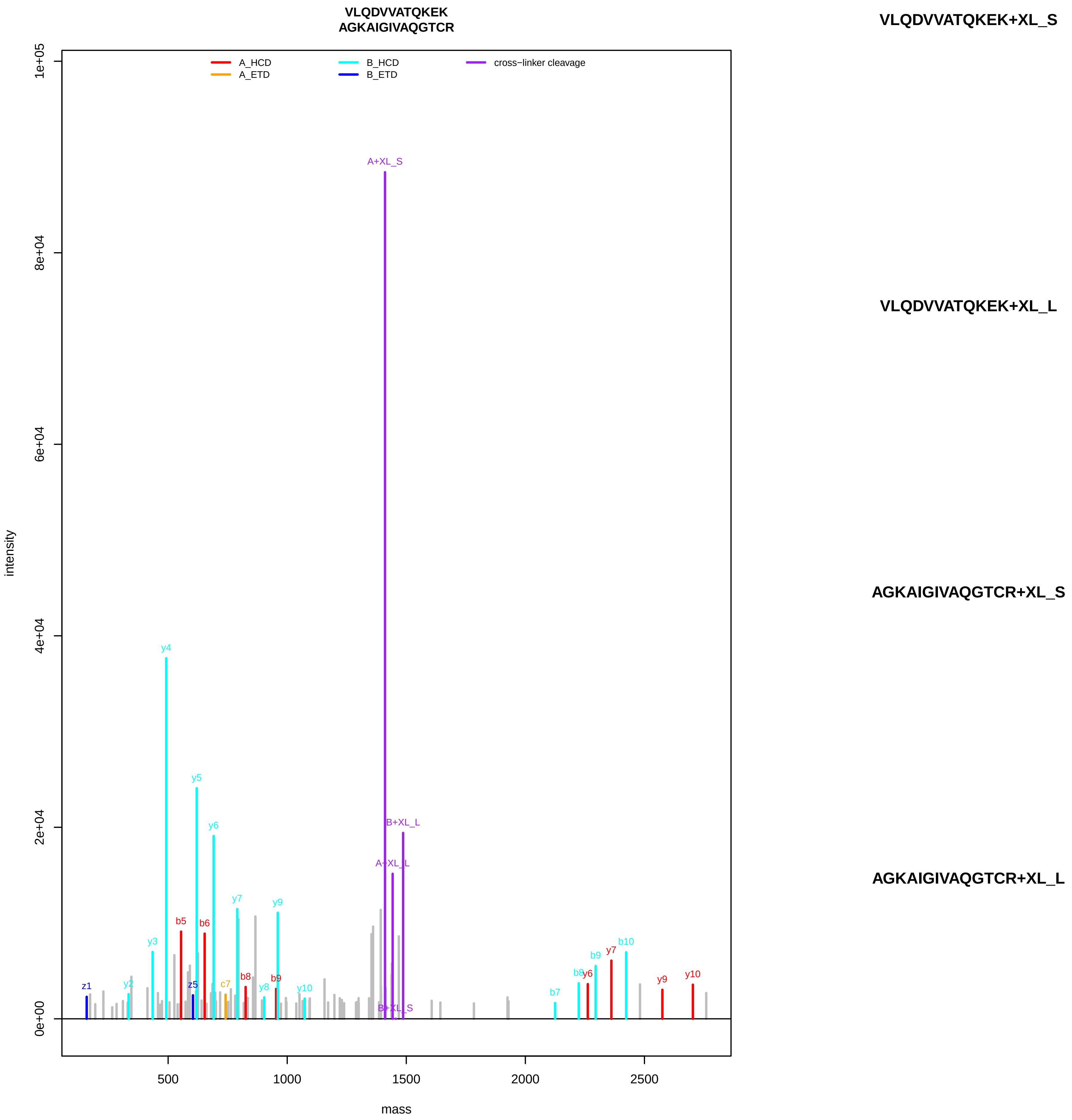

VLQDVVATQKEK
AGKAIGIVAQGTCR
A_HCD
A_ETD
B_HCD
B_ETD
cross−linker cleavage
intensity
mass
0e+00
2e+04
4e+04
6e+04
8e+04
1e+05
500
1000
1500
2000
2500
A+XL_S
B+XL_L
A+XL_L
B+XL_S
y4
y5
y6
y7
y9
y3
y2
z1
z5
b5
b6
c7
b8
b9
y8
y10
b7
b8
y6
b9
y7
b10
y9
y10
VLQDVVATQKEK+XL_S
VLQDVVATQKEK+XL_L
AGKAIGIVAQGTCR+XL_S
AGKAIGIVAQGTCR+XL_L
